# Autophagy Facilitates Antibody-Enhanced Dengue Virus Infection in Human Pre-Basophil/Mast Cells

**DOI:** 10.1371/journal.pone.0110655

**Published:** 2014-10-16

**Authors:** Yi-Ting Fang, Shu-Wen Wan, Yi-Tien Lu, Ju-Han Yao, Chiou-Feng Lin, Li-Jin Hsu, Michael G. Brown, Jean S. Marshall, Robert Anderson, Yee-Shin Lin

**Affiliations:** 1 Department of Microbiology and Immunology, National Cheng Kung University Medical College, Tainan, Taiwan; 2 Center of Infectious Disease and Signaling Research, National Cheng Kung University, Tainan, Taiwan; 3 Department of Pathology and Laboratory Medicine, Emory University School of Medicine, Atlanta, Georgia, United States of America; 4 Institute of Clinical Medicine, National Cheng Kung University Medical College, Tainan, Taiwan; 5 Department of Medical Laboratory Science and Biotechnology, National Cheng Kung University Medical College, Tainan, Taiwan; 6 Department of Microbiology and Immunology, Dalhousie University, Halifax, Canada; University of Tennessee Health Science Center, United States of America

## Abstract

**Background:**

Dengue virus (DENV) infection can cause severe hemorrhagic disease in humans. Although the pathogenic mechanisms underlying severe DENV disease remain unclear, one of the possible contributing factors is antibody-dependent enhancement (ADE) which occurs when sub-neutralizing antibodies derived from a previous DENV infection enhance viral infection through interaction between virus-antibody complexes and FcR-bearing cells, such as macrophages and basophil/mast cells. Although recent reports showed that DENV induces autophagy, the relationship between antibody-enhanced DENV infection and autophagy is not clear.

**Methodology/Principal Findings:**

We showed that sub-neutralizing antibodies derived from dengue patient sera enhanced DENV infection and autophagy in the KU812 pre-basophil-like cell line as well as the HMC-1 immature mast cell line. Antibody-enhanced DENV infection of KU812 cells increased the number of autophagosome vesicles, LC3 punctation, LC3-II accumulation, and p62 degradation over that seen in cells infected with DENV alone. The percentages of DENV envelope (E) protein-positive cells and LC3 puncta following antibody-enhanced DENV infection of KU812 cells were reduced by the autophagy inhibitor 3-MA. Antibody-enhanced DENV infection of HMC-1 cells showed co-localization of DENV E protein and dsRNA with autophagosomes, which was inhibited by 3-MA treatment. Furthermore, DENV infection and replication were reduced when KU812 cells were transfected with the autophagy-inhibiting Atg4B^C74A^ mutant.

**Conclusions/Significance:**

Our results demonstrate a significant induction of autophagy in antibody-enhanced DENV infection of pre-basophil-like KU812 and immature mast cell-like HMC-1 cells. Also, autophagy plays an important role in DENV infection and replication in these cells. Given the importance of ADE and FcR-bearing cells such as monocytes, macrophages and basophil/mast cells in dengue disease, the results provide insights into dengue pathogenesis and therapeutic means of control.

## Introduction

Dengue disease is a severe health problem in tropical or subtropical areas of the world [1], [2]. Dengue virus (DENV) infection can cause mild dengue fever to severe life-threatening dengue hemorrhagic fever and dengue shock syndrome [3]–[5]. Several mechanisms are involved in the pathogenesis of dengue disease progression, including antibody-dependent enhancement (ADE) and abnormal host immune responses [6]–[13]. ADE of DENV infection occurs when heterotypic, sub-neutralizing antibodies derived from a previous infection enhance viral uptake through the interaction between virus-antibody complexes and Fcγ receptors on Fcγ receptor-bearing cells, particularly monocytes, macrophages and basophil/mast cells [14]–[19].

Basophil/mast cells expressing Fc receptors play an important role in a wide variety of inflammatory reactions and in host defense against pathogens. These cells selectively produce and secrete numerous factors including chemokines, cytokines, lipid mediators, and granule-associated products [20]. In dengue patients, increased levels of blood and urinary histamine, a major granule product of basophil/mast cells, have been reported to correlate with disease severity [21], [22]. A large histological study of dengue hemorrhagic fever (DHF) patients noted mast cell activation as demonstrated by swelling and vacuolation of the cytoplasm, and loss of granule integrity [23].

Autophagy is an evolutionarily ancient pathway which contributes to cell survival. Conditions such as nutrient starvation, pathogen infection, and other environmental stresses can induce autophagy. During autophagy, portions of the cytoplasm or small organelles are sequestered into double-membrane vesicles called autophagosomes. Autophagosomes ultimately fuse with lysosomes to generate single-membrane vesicles termed autophagolysosomes, where the contents are subsequently degraded. Transmission electron microscopy (TEM) has been used to detect autophagosome formation, and the expression level of LC3-II is another indicator [24]. Autophagy may play both anti-viral and pro-viral roles in viral infection and pathogenesis [25]. Previous studies showed that DENV induces autophagy in human hepatoma cell lines and peripheral blood monocytes to promote viral replication [26]–[28]. A DENV nonstructural protein, NS4a, has been reported to induce autophagy to protect cells against death and enhance DENV replication [29]. In contrast, the induction of autophagy has been reported to reduce DENV output in monocytic cell line [30]. It has also been shown that DENV triggers autophagy to regulate lipid metabolism [31], [32]. A previous study by Ubol *et al*. showed that entry of DENV-antibody complexes into human monocytic cells activated negative regulators DAK and Atg5-Atg12, which then disrupted the RIG-I/MDA-5 signaling cascade and reduced type-1 interferon (IFN) production. This would lead to suppression of IFN-mediated antiviral responses [33]. Their study indicated the involvement of autophagy-related proteins Atg5-Atg12 to evade host innate immune responses. In the present study, we investigated the induction of autophagy by antibody-enhanced DENV infection of pre-basophil-like KU812 cells and immature mast cell-like HMC-1 cells. We provide evidence of a definitive link between antibody-enhanced DENV infection and autophagosome formation and elucidate mutual regulatory pathways between virus replication and induction of autophagy.

## Materials and Methods

### Cell cultures

Human KU812 basophil precursor cells [34], [35] were cultured in RPMI 1640 medium (Thermo Scientific Laboratories) containing 10 mM N-2-hydroxyethylipiperazine-N′-2-ethanesulfonic acid (HEPES) with 10% fetal bovine serum (FBS). Human HMC-1 immature mast cells [36] were cultured in IMDM medium (Life Technologies) containing 10% FBS. Baby hamster kidney cells (BHK-21) [37] and C6/36 cells [38] were cultured in Dulbecco's modified Eagles medium (DMEM) (Life Technologies) containing 10% FBS and antibiotics. Cells were cultured in 37°C in a humidified atmosphere of 5% CO_2_. For the treatment with autophagy inhibitor, cells were pre-incubated with 1 mM 3-methyladenine (3-MA; Sigma-Aldrich) for 1 h before DENV infection. 3-MA in 0.5 mM was maintained in the culture medium during the incubation period.

### Dengue virus culture

Dengue virus serotype 2 (DENV2, strain 16681) originally isolated from a Thai patient who suffered from DHF [39] was used throughout this study and was maintained in C6/36 cells. Briefly, monolayers of C6/36 cells were incubated with DENV at a multiplicity of infection (MOI) of 0.01 and incubated at 28°C in 5% CO_2_ for 5 days. The cultured medium was harvested and cell debris was removed by centrifugation at 900×*g* for 10 min. After further centrifugation at 16,000×*g* for 10 min, the virus supernatant was collected and stored at −80°C until use. Virus titer was determined by plaque assay using the BHK-21 cell line.

### Dengue patient sera

For ADE assay of DENV infection, a dengue-immune serum pool was obtained from nine convalescent-phase sera from patients recovering from DENV2 infection. Dengue-convalescent patient sera were collected in Thailand in 1990 as part of long-standing surveillance and provided by Dr. Bruce Innis (Armed Forces Research Institute of Medical Science, Bangkok, Thailand) and described previously [40].

### Dengue virus infection

Aliquots of DENV were resuspended with or without 1∶10,000 dilution of pooled dengue patient sera for 1 h at 4°C. KU812 or HMC-1 cells were incubated with DENV (with or without pooled dengue patient sera) at MOI of 1 for 90 min at 4°C. Cells were then washed twice with RPMI medium to remove unabsorbed virus and antibodies. Cells were resuspended and supplemented with 2% FBS-containing medium at 37°C for further incubation.

### Plaque assay

BHK-21 cells were plated onto 12-well plates (1×10^5^ cells/well) and cultured in DMEM under CO_2_-enriched conditions. Supernatants and cell lysates from DENV-infected cells were serially diluted and inoculated with BHK-21 cells for plaque assay. After 2 h post-infection, the solution was replaced with fresh DMEM containing 2% FBS and 0.5% methyl cellulose (Sigma-Aldrich). At five days post-infection, the medium was removed, and the cells were fixed and stained with 1% crystal violet, 0.64% NaCl, and 2% formalin (Sigma-Aldrich).

### Flow cytometry analysis

Following DENV infection, cells were washed with PBS, fixed with 1% formaldehyde, and permeabilized with 0.1% saponin (Sigma-Aldrich) at room temperature for 10 min. Fc receptors of cells were blocked with 1∶100 dilution (in permeabilizing buffer) of normal human sera (approved by the Institutional Review Board of National Cheng Kung University Hospital, No. A-ER-102-123) at 4°C for 1 h. After washing, cells were then stained with anti-DENV envelope (E) protein or anti-nonstructural protein 4B (NS4B) (GeneTex) at 4°C for 30 min. Cells were incubated with Alexa488-conjugated secondary antibody (Life Technologies) at 4°C for 30 min and analyzed using FACS Calibur (BD Biosciences). For the anti-E antibody-enhanced DENV infection experiment, cells were then stained with FITC-conjugated anti-E antibodies at 4°C for 1 h and analyzed using FACS Calibur. For the Atg4B mutant-transfected antibody-enhanced DENV infection experiment, cells were stained with anti-NS4B antibodies at 4°C for 30 min, followed by Alexa647-conjugated secondary antibody (Life Technologies) at 4°C for 30 min, and analyzed using an LSRFortessa instrument (BD Biosciences).

### Immunofluorescence

Cells were fixed with 1% formaldehyde (Sigma-Aldrich), permeabilized with 0.1% saponin, and then blocked Fc receptors with normal human sera. Cells were then stained with anti-E, anti-double strand RNA (dsRNA) (English & Scientific Consulting), or anti-LC3 (MBL) antibodies at 4°C for overnight. Cells were then incubated with Alexa488- or Alexa594-conjugated secondary antibody (Life Technologies) at 4°C for 1 h and analyzed with a FV1000 confocal microscope (Olympus).

### Cell transfection

The plasmids (mStrawberry and mStrawberry-Atg4B^C74A^) were gifts of Dr. Tamotsu Yoshimori (Osaka University, Japan). Briefly, the Atg4B cDNA was cloned from genomic DNA isolated from mouse embryonic fibroblast (MEF) cells and was inserted into pmStrawberry-C1 using engineered BamHI and KpnI sites; the point mutation was introduced using the QuikChange Site-Directed mutagenesis system (Stratagene) [41]. For cell transfection, KU812 cells (1×10^7^ cells) were resuspended in 0.6 ml RPMI-1640 medium containing 10 mg/ml BSA, and mixed with 20 µg plasmid DNA in electroporation cuvette (0.4-cm electrode gap) and subjected to 220 V for 70 msec by Gene Pulser II Electroporation System (Bio-Rad). After electroporation, the cells were washed with fresh medium and then cultured for 48 h before DENV infection.

### Transmission electron microscopy

Twenty-four h after DENV infection, KU812 cells were fixed with 2.5% glutaraldehyde (Sigma-Aldrich) in 0.1 M cacodylate buffer containing 4% sucrose, 1 mM MgCl_2_ and 1 mM CaCl_2_, and post-fixed in 1% osmium tetroxide (Sigma-Aldrich). The cells were further dehydrated with ethanol and embedded with LR White. Ultrathin sections were stained with uranyl acetate and lead citrate (Sigma-Aldrich), and then observed using a HT7000 transmission electron microscope (Hitachi).

### Western blot analysis

Following infection with DENV, cells were lysed using a Triton X-100 based lysis buffer with a protease inhibitor mix and phosphatase inhibitors (Sigma-Aldrich). Cell extract was separated using SDS-PAGE and transferred to polyvinylidene difluoride membrane (Millipore). After blocking with 5% nonfat milk in PBS-T (0.05% Tween-20), blots were probed with anti-LC3, -NS4B, -p62 (MBL), and -β-actin (Santa Cruz Biotechnology) antibodies at 4°C for overnight. After washing with PBS-T, blots were treated with horseradish peroxidase (HRP)-conjugated secondary antibodies (Cell Signaling Technology) in 1∶5000 dilution at room temperature for 1 h. Blots were developed using western lightning chemiluminescence reagent (Perkin Elmer for all blots, except Millipore for LC3 blot).

### Statistics

We used the paired *t*-test for statistical analysis. Statistical significance was set at *P*<0.05.

## Results

### Antibody-enhanced DENV infection and autophagosome formation in KU812 cells

**Figure 1:**
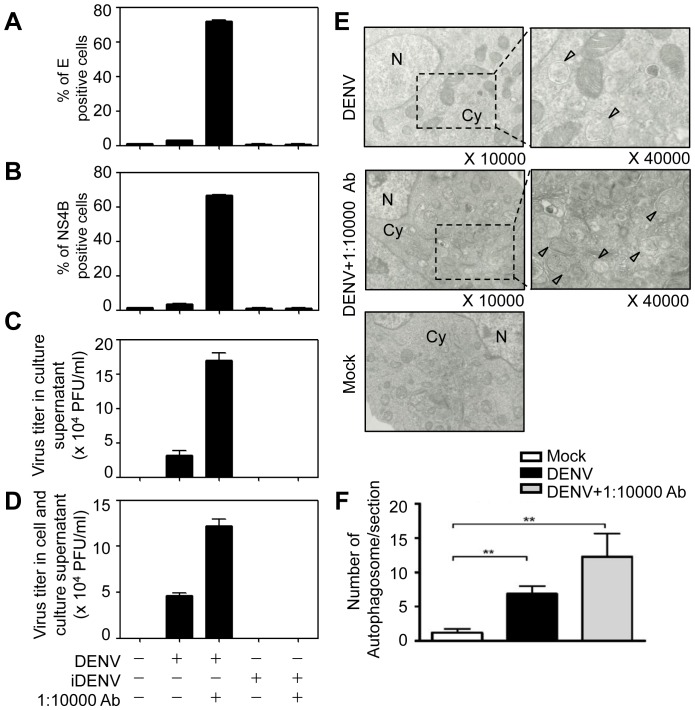
Antibody-enhanced DENV infection and autophagosome formation in KU812 cells. (A and B) Cells were inoculated with medium alone (Mock), with DENV alone (MOI = 1), or with DENV in the presence of a sub-neutralizing dilution (1∶10,000) of dengue patient sera at 4°C for 1.5 h. After washing, cells were resuspended in fresh medium and incubated at 37°C for 24 h. The DENV E protein (A) and NS4B protein (B) were detected by flow cytometry. Infection of UV-inactivated DENV (iDENV) was served as negative control. The means of three independent experiments ± SD are shown. (C and D) After 24 h post-infection, the culture supernatant (C) and the mixture of cell and culture supernatant (D) were collected to determine viral titers by plaque assay. The means ± SD of three independent experiments are shown. (E) After infection for 24 h, cells were fixed and observed under TEM. We analyzed five cells by TEM in each condition including mock, DENV alone, and ADE. One section per cell was obtained to quantify the autophagosome vesicles. The black square areas in the left panels were amplified (×40000) and shown in right panels. The arrowheads indicate the autophagosomes. Cy: cytoplasm; N: nucleus. (F) The quantification of autophagosome numbers in each section from (E) is shown. The means ± SD of three independent experiments are shown. **P<0.01.

Recent reports demonstrated that autophagy is observed in several kinds of DENV-infected cells [42]. However, the relationship between antibody-enhanced DENV infection and autophagy is still unclear. Human pre-basophil-like KU812 cells were inoculated with DENV (MOI = 1) in the presence or absence of dengue patient sera (1∶10,000 dilution). Detection of DENV infection in KU812 cells was performed by flow cytometry (Figure 1A and 1B) and plaque assay (Figure 1C and 1D). Our previous study indicated very few virus-positive cells when KU812 cells were inoculated with DENV in combination with normal (i.e. dengue non-immune) human sera [43]. In addition, although the possibility of non-specific effect of antibody-containing serum to other RNA viruses cannot be completely ruled out, it was previously reported that infection of KU812 cells with another virus (RSV) was not enhanced by human RSV-positive, dengue-negative sera [43]. We herein showed the intracellular expression of E protein and NS4B protein at 24 h post-infection was significantly enhanced by antibody-enhanced DENV infection (Figure 1A and 1B). In addition, virus titers from the supernatant of antibody-enhanced DENV-infected KU812 cells (Figure 1C) and the combined supernatant and infected cells (Figure 1D) were increased than those from KU812 cells infected with DENV alone. UV-inactivated DENV (iDENV) served as negative control (Figure 1A-1D). Furthermore, we found that antibody-enhanced DENV infection of KU812 cells increased autophagosome formation as observed by electron microscopy (Figure 1E, arrowhead). The number of autophagosomes from one section in each cell was quantified from Figure 1E and shown in Figure 1F.

**Figure 2:**
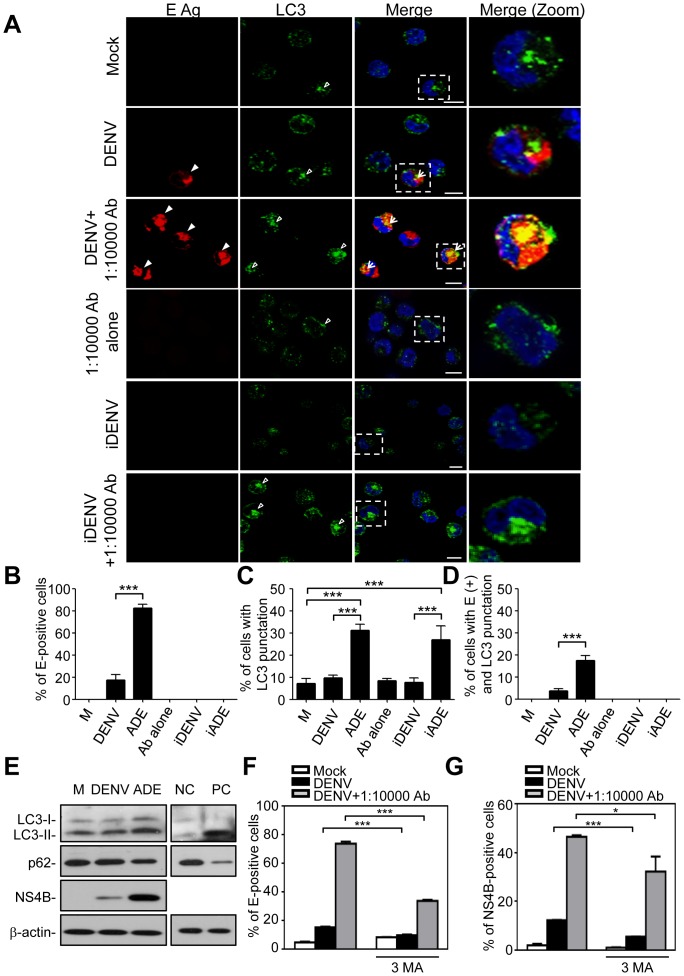
Co-localization of DENV E protein and LC3 punctation in antibody-enhanced DENV infection of KU812 cells. (A) KU812 cells were incubated with medium alone (Mock), with DENV alone, with DENV in the presence of sub-neutralizing dengue patient sera, sub-neutralizing dengue patient sera alone, with UV-inactivated DENV (iDENV) alone, or iDENV in the presence of sub-neutralizing dengue patient sera. After infection, cells were fixed, permeabilized, and stained with anti-DENV E protein (red), anti-LC3 (green), and DAPI (blue). Cells were then mounted and observed by confocal microscopy. The square areas are zoomed-in images and shown in the right panels (merge, zoom). Bar: 10 µm. The imaging data were repeated three times and one set of representative results is shown. (B) The quantification of E-positive cells (A, filled arrowheads) is shown. The means ± SD of three independent experiments are shown. ***P<0.005. (C) The quantification of LC3 punctation cells (A, empty arrowheads) is shown. The means ± SD of three independent experiments are shown. ***P<0.005. (D) The percentages of cells with E-positive and LC3 punctation (A, arrows) are shown. The means ± SD of three independent experiments are shown. ***P<0.005. (E) After 24 h post-infection, the protein levels of LC3, p62, and NS4B from total cell lysates were detected by Western blotting. β-actin served as internal control. NC: negative control (nutrient-rich medium); PC: positive control (starvation; Hank's balanced salt solution). (F and G) KU812 cells were pre-treated with or without 5 mM 3-MA for 1 h before incubation with medium alone (Mock), DENV alone, or DENV with sub-neutralizing dengue patient sera. 3-MA was maintained in the medium during DENV infection. After 24 h post-infection, the expression of DENV E protein (F) and NS4B protein (G) was detected by flow cytometry. The means ± SD of three independent experiments are shown. **P<0.01, ***P<0.005.

We further confirmed autophagy in DENV-infected KU812 cells using confocal microscopy to analyze the expression of DENV E protein and LC3 punctation, as a marker for autophagy. In addition to increased DENV E protein expression (Figure 2A, filled arrowhead), antibody-enhanced DENV infection also increased LC3 punctation (Figure 2A, empty arrowhead). Furthermore, KU812 cells were infected with iDENV in the presence and absence of subneutralizing dengue patient sera. The LC3 punctation also apparently increased in the iDENV infection of KUB12 cells with subneutralizing dengue patient sera (Figure 2A). The co-localization of E-protein and LC3 punctation is also shown (merge, zoom, yellow). The quantified results of E protein expression and LC3 punctation from Figure 2A are shown in Figure 2B and Figure 2C, respectively. Not every cell expressing E protein showed LC3 punctation and vice versa. We therefore further analyzed the percentages of cells with both E protein expression and LC3 punctation (Figure 2A, merge, arrow, and Figure 2D). Cell lysates collected from cells infected with DENV alone or antibody-enhanced DENV were analyzed by Western blotting to confirm autophagy induction and viral infection. LC3-II accumulation and p62 degradation as autophagy indicators as well as DENV NS4B expression were increased in the antibody-enhanced DENV-infected cells (Figure 2E). In addition, nutrient-rich medium incubation served as a negative control and stimulation of autophagy by starvation served as a positive control of autophagy (Figure 2E). It has been previously demonstrated that dsRNA, as an indicator of DENV replication, can co-localize with LC3 punctation structures in hepatocytes [28]. Here, we found that the number of dsRNA-positive KU812 cells (Figure S1, filled arrowhead) as well as LC3 puncta (Figure S1, empty arrowhead) were increased in antibody-enhanced, compared to DENV alone, infection. Also, numbers of dsRNA/LC3 punctation-positive KU812 cells were increased in antibody-enhanced DENV infection (Figure S1, merge, arrow). The co-localization of dsRNA and LC3 punctation is also shown (Figure S1, zoom, yellow). It is noteworthy that co-localization of dsRNA and LC3 punctation occurs sporadically, indicating either a transient interaction or an interaction involving only a subset of the total dsRNA. Furthermore, the autophagy inhibitor 3-MA was used to assess if modulation of autophagy may alter DENV infection. KU812 cells were infected with DENV alone or in combination with enhancing antibody and with or without 3-MA. Results showed that treatment with 3-MA inhibited DENV E and NS4B protein expression in KU812 cells whether they were inoculated with DENV alone or with enhancing antibodies (Figure 2F and 2G). A representative histogram of each group is shown in Figure S2.

**Figure 3:**
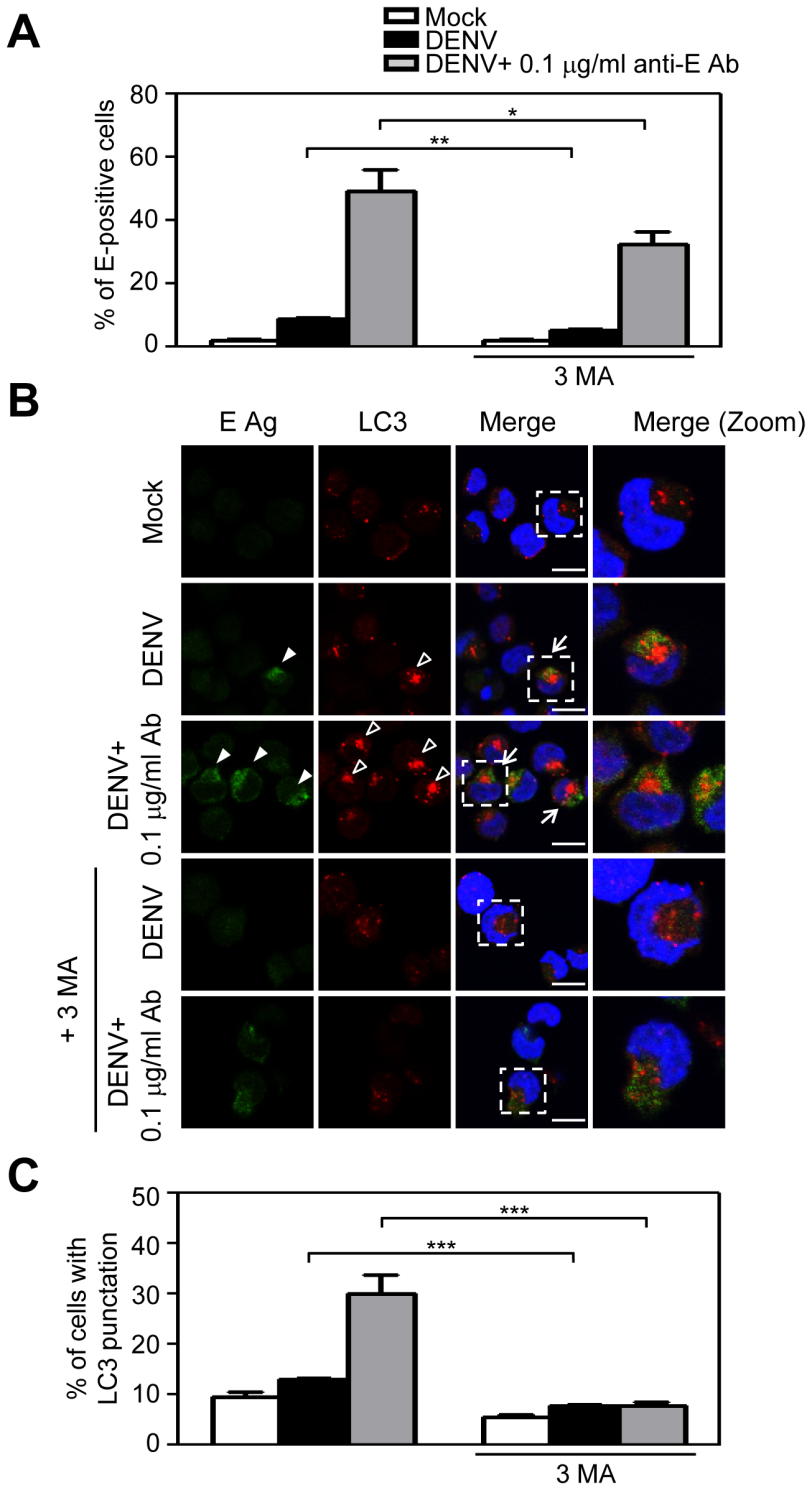
Sub-neutralizing anti-E monoclonal antibody enhances DENV infection and induces autophagy in KU812 cells. KU812 cells were pretreated with or without 5 mM 3-MA for 1 h before incubation with medium alone (Mock), DENV alone, or DENV with sub-neutralizing anti-E. 3-MA was maintained in the medium during DENV infection. (A) After 24 h post-infection, the expression of DENV E protein was detected by flow cytometry. The means ± SD of three independent experiments are shown. *P<0.05, **P<0.01. (B) Cells were fixed, permeabilized, and stained with anti-DENV E protein (green), anti-LC3 (red), and DAPI (blue). Cells were then mounted and observed by confocal microscopy. The square areas are zoomed-in images and shown in the right panels (merge, zoom). Bar: 10 µm. The imaging data were repeated three times and one set of representative results is shown. (C) The quantification of LC3 punctation cells (B, empty arrowheads) is shown. The means ± SD of three independent experiments are shown. ***P<0.005.

We further used purified anti-DENV E protein monoclonal antibody 137-22 (a gift from Dr. Huan-Yao Lei's laboratory) [44] at a sub-neutralizing level, and results showed that the percentages of E-positive cells were significantly enhanced by DENV infection in the presence of 0.1 µg/ml purified anti-E monoclonal antibody over that seen in cells infected with DENV alone (Figure 3A). Furthermore, the percentages of E-positive cells were reduced by 3-MA treatment following DENV infection with or without sub-neutralizing anti-E antibody (Figure 3A). The confocal microscopy results also showed anti-E antibody-enhanced DENV infection (Figure 3B, filled arrowhead). Moreover, LC3 punctation was induced by DENV infection in the presence of sub-neutralizing anti-E antibody (Figure 3B, empty arrowhead). Some cells expressed both E protein and LC3 punctation (Figure 3B, arrow). LC3 punctation was also reduced by 3-MA treatment (Figure 3B). The quantified results are shown in Figure 3C.

### Co-localization of DENV E protein and dsRNA with autophagosomes in HMC-1 cells infected with DENV with or without enhancing antibody

**Figure 4:**
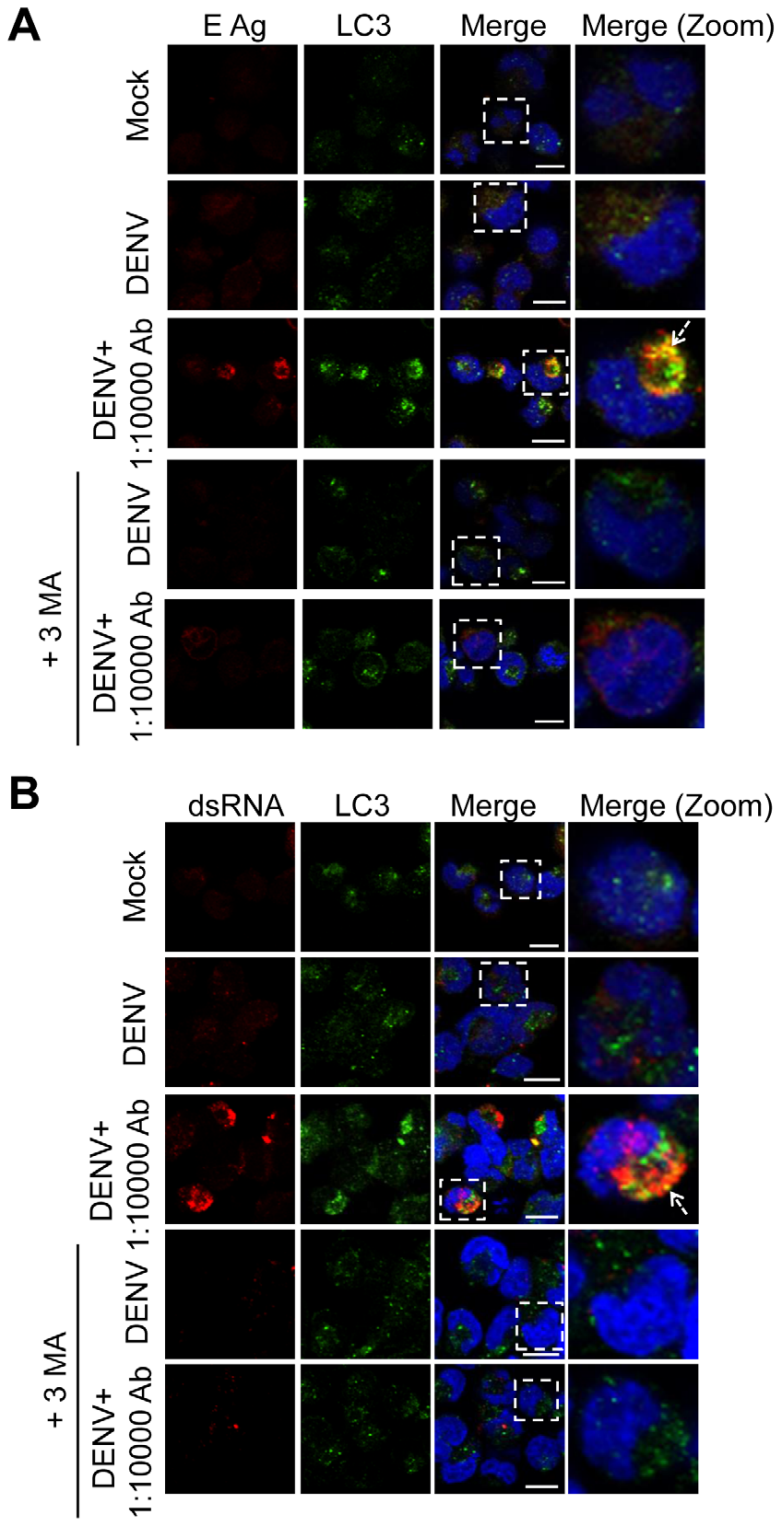
Co-localization of DENV E protein and dsRNA with autophagosome in HMC-1 cells after infection with DENV alone or with enhancing antibody. HMC-1 cells were pretreated with or without 1 mM 3-MA for 1 h. Cells were then incubated with medium alone (Mock), with DENV (MOI = 1), or with DENV in the presence of sub-neutralizing dengue patient sera with or without 0.5 mM 3-MA during the incubation periods. (A) After 24 h post-infection, cells were fixed, permeabilized, and stained with anti-E protein (red), LC3 protein (green), and DAPI (blue). The imaging data were repeated two times and one set of representative results is shown. (B) After 24 h post-infection, cells were fixed, permeabilized, and stained for anti-double strand (ds) RNA (red), LC3 protein (green), and DAPI (blue). After mounting, cells were observed by confocal microscopy. The square areas are zoomed-in images and shown in the right panels (merge, zoom). Bar: 10 µm. The imaging data were repeated three times and one set of representative results is shown.

We further confirmed autophagy and DENV infection in the immature mast cell line HMC-1. After 24 h post-infection of DENV alone (MOI = 1), few HMC-1 cells expressed E protein and LC3 punctation. In contrast, antibody-enhanced DENV infection of HMC-1 cells resulted in increased levels of E protein and LC3 punctation (Figure 4A). Co-localization of E proteins with LC3 punctation was also observed in some (dotted arrow), but not all, HMC-1 cells. When HMC-1 cells were treated with autophagy inhibitor 3-MA, the expression of E and LC3 punctation and their co-localization were inhibited (Figure 4A). Similar findings were observed in the expression of dsRNA and its co-localization with LC3 punctation following antibody-enhanced DENV infection of HMC-1 cells (Figure 4B). The enhanced expression of dsRNA and its co-localization with LC3 punctation were also inhibited when cells were treated with 3-MA (Figure 4B).

### Autophagy facilitates DENV infection and antibody-enhanced DENV infection in KU812 cells

**Figure 5:**
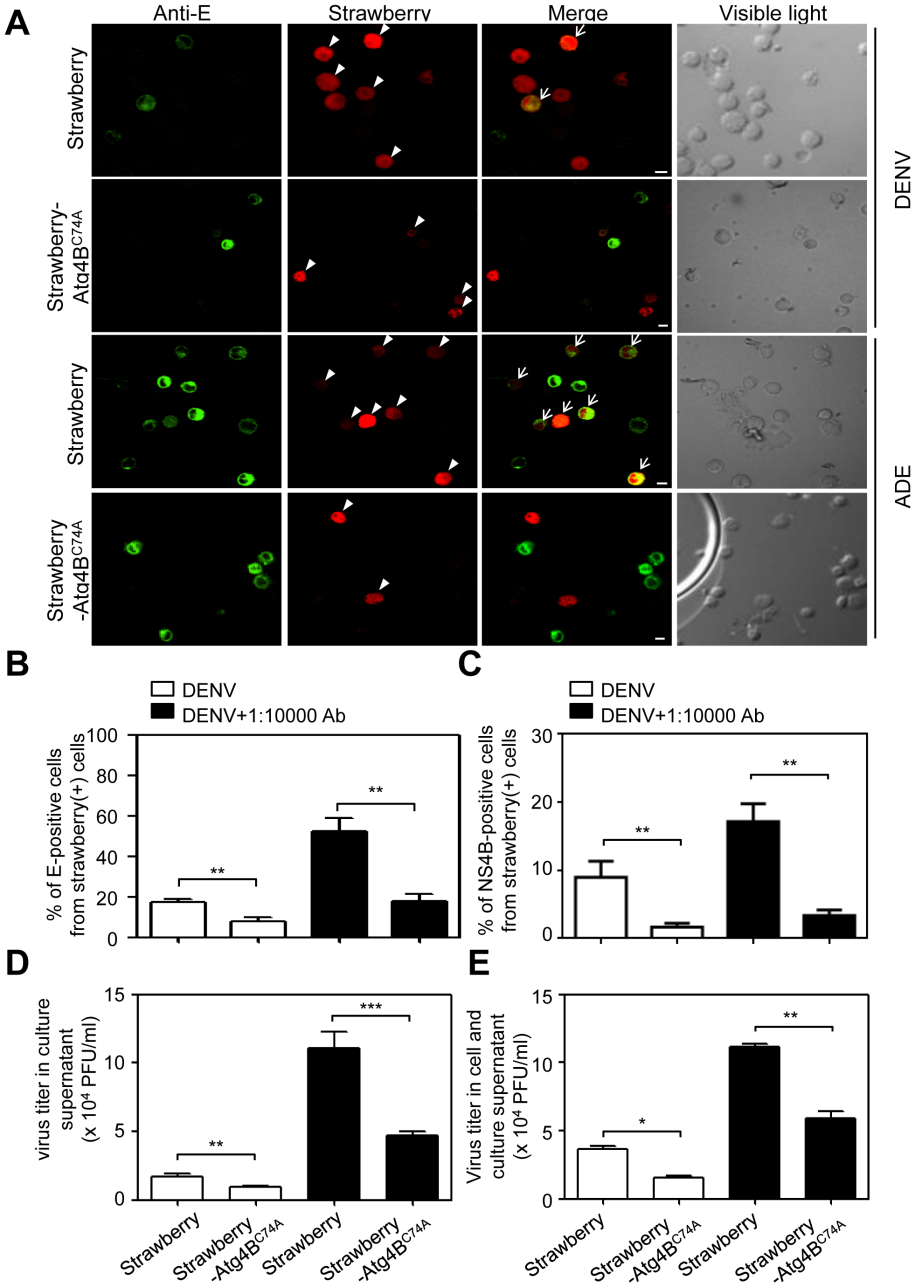
Inhibition of autophagy reduces DENV infection and virus titer after DENV infection with or without enhancing antibody. (A) KU812 cells were transfected with strawberry or strawberry-Atg4B^C74A^ plasmids. After transfection and incubation for 48 h, strawberry- and strawberry-Atg4B^C74A^-expressing KU812 cells were infected with DENV or DENV with sub-neutralizing dengue patient sera. After 24 h post-infection, cells were fixed, permeabilized, stained with anti-E (green), and observed by confocal microscopy. The arrowheads indicate the strawberry- and strawberry-Atg4B^C74A^-expressing cells (red). The arrows (merge) indicate the cells which possess both green and red fluorescence. The visible images of total cells are shown in the right panels. The imaging data were repeated three times and one set of representative results is shown. (B) The number of E-positive cells was counted from 200 red cells from Fig. 5A. The percentage of E-positive cells from red cells was then quantified. The means ± SD of three independent experiments are shown. (C) KU812 cells were transfected with strawberry or strawberry-Atg4B^C74A^ plasmids. After transfection and incubation for 48 h, strawberry- and strawberry-Atg4B^C74A^-expressing KU812 cells were infected with DENV or DENV with sub-neutralizing dengue patient sera. After 24 h post-infection, cells were fixed, permeabilized, stained with anti-NS4B, and followed by Alexa647-conjugated secondary antibody. The percentage of NS4B-positive cells in red cells was then determined by flow cytometry. The means ± SD of three independent experiments are shown. (D and E) After 24 h post-infection, the culture supernatant (D) and the mixture of cell and culture supernatant (E) were collected to detect the viral titers by plaque assay. The means ± SD of three independent experiments are shown. *P<0.05, **P<0.01, ***P<0.005.

In order to further confirm the role of autophagy, we established the Atg4B^C74A^-expressing KU812 cells. Atg4B^C74A^ is an inactive Atg4B mutant due to the mutation of the catalytic cysteine residue (Cys74) and in consequence lacks protease activity. Atg4B^C74A^ can hamper autophagosome closure and has been utilized as a useful tool to inhibit autophagy [45]. Cells transfected with empty strawberry plasmids served as control. We first confirmed that LC3 punctation was inhibited in mutant Atg4B^C74A^-expressing cells after transfection with Strawberry-Atg4B^C74A^ plasmids in nutrient-rich and starvation conditions (Figure S3). Since not all cells were successfully transfected with Strawberry (transfection efficiency 18.3%) or Strawberry-Atg4B^C74A^ plasmids (transfection efficiency 14.1%), we analyzed the infectivity only from the cells showing red fluorescence. We counted the same numbers of Strawberry- and Strawberry-Atg4B^C74A^-expressing cells (red fluorescence; Figure 5A, arrowhead) to further determine the DENV-infected cells, which expressed DENV E protein (green fluorescence; Figure 5A, merge, arrow). Quantified results from Figure 5A showed that the percentage of E-positive cells was increased in antibody-enhanced strawberry-expressing cells (Figure 5B). Besides E protein, we also confirmed that the percentage of NS4B-positive cells was increased in antibody-enhanced strawberry-expressing cells by flow cytometry (Figure 5C). Furthermore, in both strawberry-Atg4B^C74A^-expressing cells infected with DENV alone or with enhancing antibody, the percentages of E and NS4B-positive cells were inhibited (Figure 5B and 5C). Reduced viral titers were also found in culture supernatants (Figure 5D) and the combined supernatant and lysates from strawberry-Atg4B^C74A^-expressing cells (Figure 5E). Since LC3 is also involved in the autophagosome formation [24], we also verified the role of LC3 in DENV infection. Cells were transfected with plasmid expressing shLuc (control) or shLC3. Successful knock-down of shLC3 expression was demonstrated by Western blot analysis (Figure S4A). DENV infection of KU812 cells as determined by viral NS4B protein expression was also decreased when cells were transfected with shLC3 as compared with cells treated with control shLuc (Figure S4B and S4C). Taken together, the results indicate that inhibition of autophagy reduces DENV infection and replication in KU812 cells whether inoculated with DENV alone or with enhancing antibody.

## Discussion

Previous reports have noted that DENV infection is associated with the autophagic response. DENV infection can induce ER stress [46], and ER stress has been demonstrated to activate autophagy [47]. In addition, DENV and antibody binds to FcR and activates DAK and Atg5-Atg12, which act as negative regulators to disrupt RIG-I/MDA-5 signaling and consequently type-1 IFN production [33], [48]. Several pathogens, such as poliovirus and rhinovirus, employ autophagy to evade host immune responses [49]. DENV utilizes components of the cellular autophagy pathway for its own growth benefit [26]–[28], [31]. A recent study using a novel autophagy inhibitor spautin-1 showed a direct effect of constituents of the autophagy pathway on DENV assembly [50]. Taken together, these findings suggest that pharmacological inhibition of autophagy as therapeutics against DENV infection and pathogenesis may be promising. In the present study, we showed that DENV replication and viral yield were significantly decreased when pre-basophil-like cells were treated with autophagy inhibitor 3-MA or overexpression of the mutant protein Atg4B^C74A^. DENV also utilizes autophagosomes for replication in human hepatoma cell lines [26]–[28], [51]. While autophagy was induced in response to ADE-mediated DENV2 infection of monocytic U937 cells, biochemical induction of autophagy actually resulted in a decreased virus output and downregulation of autophagy caused only slight increase in DENV levels, suggesting that autophagy may not play a significant role of DENV replication in monocytic cells [30]. Therefore, there are probably cell-type differences in the importance of DENV–autophagy interaction [30].

Although the autophagosome may be the site for DENV replication, our results showed that DENV E proteins and dsRNA co-localized with autophagosomes only in some DENV-infected pre-basophil-like cells. This finding suggests that autophagy may also indirectly promote DENV replication in these cells. Nevertheless, we cannot exclude the possibility of limited detection sensitivity in our immunofluorescence staining assay. It was previously shown that GFP-LC3 puncta did not co-localize with DENV replication markers [31]. In contrast, other studies showed the co-localization of DENV and autophagosomes, and this was observed at least 24 h post-infection [27], [28], [30]. Using a single-virus particle tracking technology to determine whether DENV interacts with autophagy machinery, Chu *et al*. showed the co-localization of DENV and LC3 puncta. They found that viral particles interacted directly with autophagosomes at an early stage of DENV infection in a hepatoma cell line and promoted DENV replication [51].

Heaton *et al.* demonstrated that DENV-induced autophagic degradation modulates cellular lipid metabolism which indirectly promotes DENV replication in infected cells [52]. Because metabolism of lipid droplets is important for mast cell differentiation and activation to further release inflammatory mediators [53], it is worthwhile to further explore whether autophagic degradation of cellular lipid droplets is involved in the promotion of DENV replication. On the other hand, we also observed that some cells show autophagy-related LC3 punctuation despite the absence of detectable DENV infection. It has been demonstrated that inflammatory cytokines IFN-γ or MIF can initiate an intracellular autophagic response [54]–[56]. Because the production of IFN-γ and MIF is also associated with DENV disease, these and/or other DENV-associated cytokines may play a role in triggering the autophagy response in non-infected cells.

Ushio *et al*. suggested that autophagy plays a crucial role in degranulation of mast cells [57]. In this study, we have proposed a novel role for autophagy that may explain immune-mediated pathology by DENV. In dengue patients, serum and urinary levels of histamine, a major granule component of basophil/mast cells, were increased and correlated with disease severity [22]. DHF and dengue shock syndrome patients also show increased serum levels of anaphylatoxins C3a, C5a, and IgE, which can activate basophil/mast cells and contribute to vascular leakage possibly leading to shock [58]. *In vitro* studies also showed that DENV-infected basophil/mast cells can release potent vasoactive cytokines, such as IL-1β and IL-6, and chemokines, such as CCL3, CCL4, and CCL5 [15], [16], [43]. DENV-induced basophil/mast cell activation elevated systemic vasoactive products including leukotrines and chymase, which may contribute to vascular leakage during DENV infection [58]. Basophil/mast cells may also play a protective role in the host response to DENV because blocking these cells or their mediators has been reported to reduce DENV clearance [59], [60]. The role of basophil/mast cells in DENV pathogenesis is still not fully defined and needs to be further investigated.

In DENV-endemic areas of the world, pre-existing sub-neutralizing antibodies may promote DENV infection via facilitating viral binding and entry into FcR-bearing cells by the mechanism of ADE [61], [62]. Besides an extracellular or extrinsic role of enhancing antibodies, ADE may also enhance infection intrinsically by regulating the production of inflammatory-associated cytokines, such as IL-6, IL-10, IL-12, TNF-α and IFN-γ [63], [64]. ADE may also disrupt innate pathways of anti-viral response [33], [48]. In basophil/mast cells, the potent vasoactive cytokines, IL-1β and IL-6, and chemokines, CCL3, CCL4, and CCL5 are increasingly released after DENV infection with enhancing antibodies [15], [16], [43]. The release of vasoactive cytokines and chemokines causing vascular leakage on a systemic level can contribute to DENV pathogenesis [65].

So far, it is still unclear what mechanism regulates the induction of autophagy in ADE-mediated DENV infection. We found that autophagy was still induced when cells were infected with UV-inactivated DENV in the presence of sub-neutralizing antibodies (Fig. 2A and 2C). This result suggests that ligation of Fc receptor with complex of DENV and enhancing antibodies may activate Fc receptor signaling and trigger the autophagy response. Therefore, the role of Fc receptor signaling in inducing the autophagy response during antibody-enhanced DENV infection needs to be further explored.

In addition to autophagy, basophil/mast cells exposed to antibody-enhanced DENV infection also trigger apoptosis [18]. DENV infection alone may also trigger apoptosis in murine and human monocytes or neurons [66]–[68]. However, other reports showed that DENV-infected cells are protected from cell death. Furthermore, flavivirus NS4A-induced autophagy protects cells against cell death and enhances viral replication [29]. The mechanisms by which apoptosis and autophagy are regulated in antibody-enhanced DENV-infection are intriguing and require further investigation.

In conclusion, several mechanisms have been previously proposed for ADE in dengue disease pathogenesis including promoting viral entry into FcR-bearing cells, the induction of IL-10 to inhibit the IFN-mediated anti-viral pathway, and the activation of DAK and Atg5-Atg12 to inhibit IFN production [33], [48]. In the present study, we demonstrate an additional mechanism in which autophagy is induced in antibody-enhanced DENV infection and leads to increased viral replication in pre-basophil-like cells. Our finding clearly links autophagy and antibody-enhanced DENV infection. Given the importance of ADE and FcR-bearing cells in dengue disease, the results of this study may provide insights into dengue pathogenesis and therapeutic strategies.

## Supporting Information

Figure S1 **Co-localization of dsRNA with autophagosomes in KU812 cells after infection with DENV alone or with enhancing antibody.** KU812 cells were incubated with medium alone (Mock), with DENV alone, or with DENV in the presence of sub-neutralizing dengue patient sera. After 24 h post-infection, cells were fixed, permeabilized, and stained with anti-double strand (ds) RNA (red), LC3 protein (green), and DAPI (blue). Cells were then mounted and observed by confocal microscopy. The filled arrowheads indicate the dsRNA-positive cells, the empty arrowheads indicate the cells with LC-3 punctation, and the arrows indicate the cells in which dsRNA is co-localized with LC3 punctation. The square areas are zoomed-in images and shown in the right panels (merge, zoom). Bar: 10 µm. The imaging data were repeated three times and one set of representative results is shown.
(TIF)

Figure S2 **The autophagy inhibitor 3-MA reduces DENV infection.** KU812 cells were pre-treated with or without 5 mM 3-MA for 1 h before incubation with medium alone (Mock), DENV alone, or DENV with sub-neutralizing dengue patient sera. 3-MA was maintained in the medium during DENV infection. After 24 h post-infection, the expression of DENV E protein and NS4B protein was detected by flow cytometry. A representative histogram of each group is shown.
(TIF)

Figure S3 **Autophagy is inhibited in the strawberry-Atg4B^C74A^-expressing KU812 cells.** (A) KU812 cells were transfected with strawberry or strawberry-Atg4B^C74A^ plasmids. After transfection and incubation for 48 h, strawberry- and strawberry-Atg4B^C74A^-expressing KU812 cells were incubated in the nutrient-rich medium or Hank's balanced salt solution (starvation). After 3 h, cells were fixed, permeabilized, stained, and observed by confocal microscopy. The filled arrowheads indicate the strawberry- and strawberry-Atg4B^C74A^-expressing cells (red). The empty arrowheads indicate LC3 punctation (green). The arrows indicate the cells which possess both green and red fluorescence. The imaging data were repeated two times and one set of representative results is shown. Bar: 20 µm (B) The percentage of LC3 punctation from red cells was quantified from two independent experiments.
(TIF)

Figure S4 **Blockade of LC3 reduces DENV infection.** KU812 cells were transfected with shRNA specifically targeting luciferase (shLuc) or LC3 (shLC3). The targeting sequence on luciferase is 5′-GCGCCATTCTATCCGCTGGAA-3′ and the targeting sequence on LC3 is 5′-CGCTTACAGCTCAATGCTAAT-3′. (A) The knockdown efficiency of LC3 in the cells of each group was shown using Western blot analysis. (B) After transfection and incubation for 48 h, cells were infected with DENV alone or DENV with sub-neutralizing dengue patient sera. After 24 h post-infection, the expression of DENV NS4B protein was detected by flow cytometry. A representative histogram of each group is also shown in the right panel.
(TIF)
