# Supplementary figures and images for: Autophagy Facilitates Antibody-Enhanced Dengue Virus Infection in Human Pre-Basophil/Mast Cells
Source: PLoS One. 2014 Oct 16;9(10):e110655. doi: 10.1371/journal.pone.0110655 (PMC4199741; doi:10.1371/journal.pone.0110655)

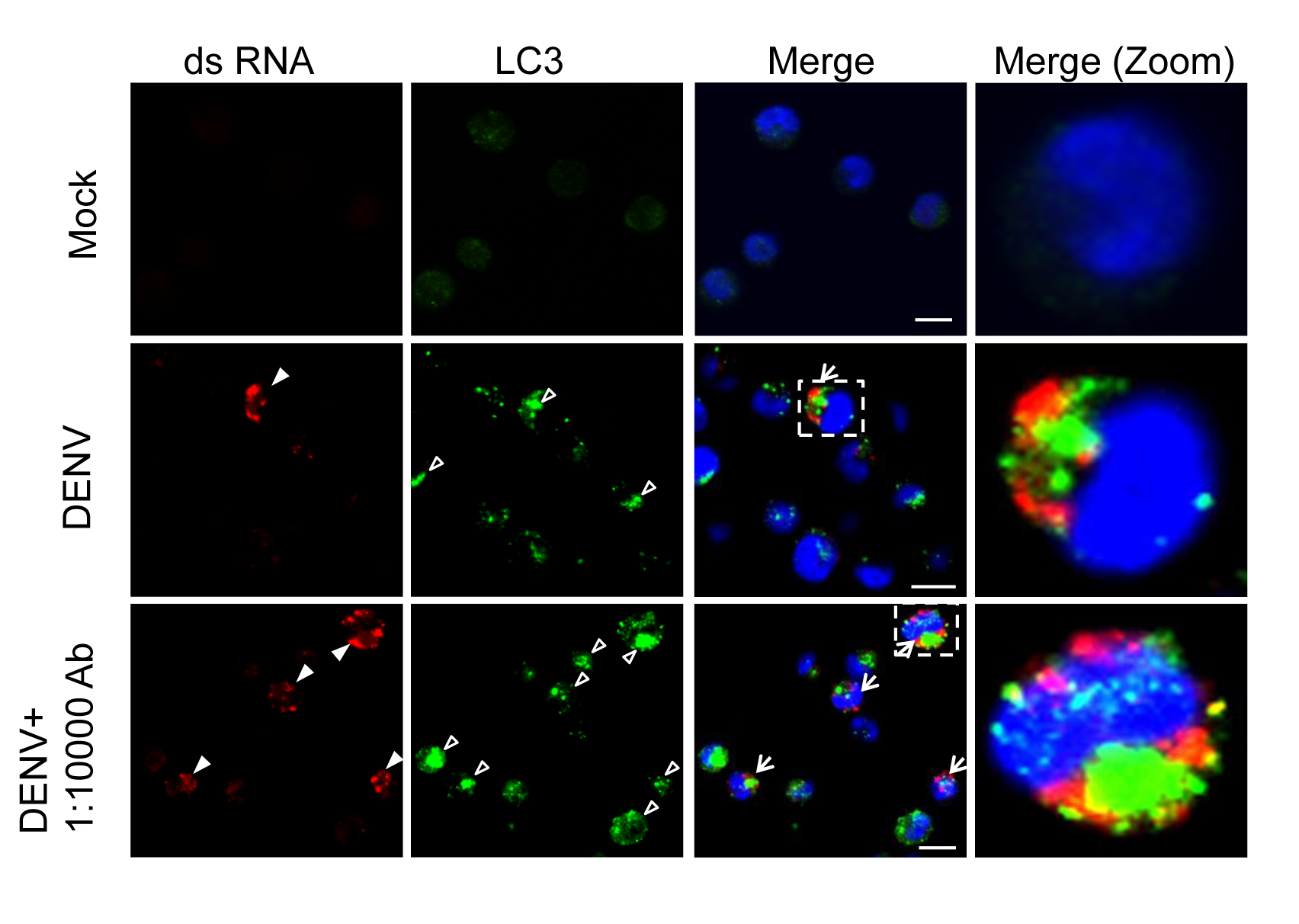

Supplement: Figure S1 — Co-localization of dsRNA with autophagosomes in KU812 cells after infection with DENV alone or with enhancing antibody. KU812 cells were incubated with medium alone (Mock), with DENV alone, or with DENV in the presence of sub-neutralizing dengue patient sera. After 24 h post-infection, cells were fixed, permeabilized, and stained with anti-double strand (ds) RNA (red), LC3 protein (green), and DAPI (blue). Cells were then mounted and observed by confocal microscopy. The filled arrowheads indicate the dsRNA-positive cells, the empty arrowheads indicate the cells with LC-3 punctation, and the arrows indicate the cells in which dsRNA is co-localized with LC3 punctation. The square areas are zoomed-in images and shown in the right panels (merge, zoom). Bar: 10 µm. The imaging data were repeated three times and one set of representative results is shown. (TIF) [file pone.0110655.s001.tif]

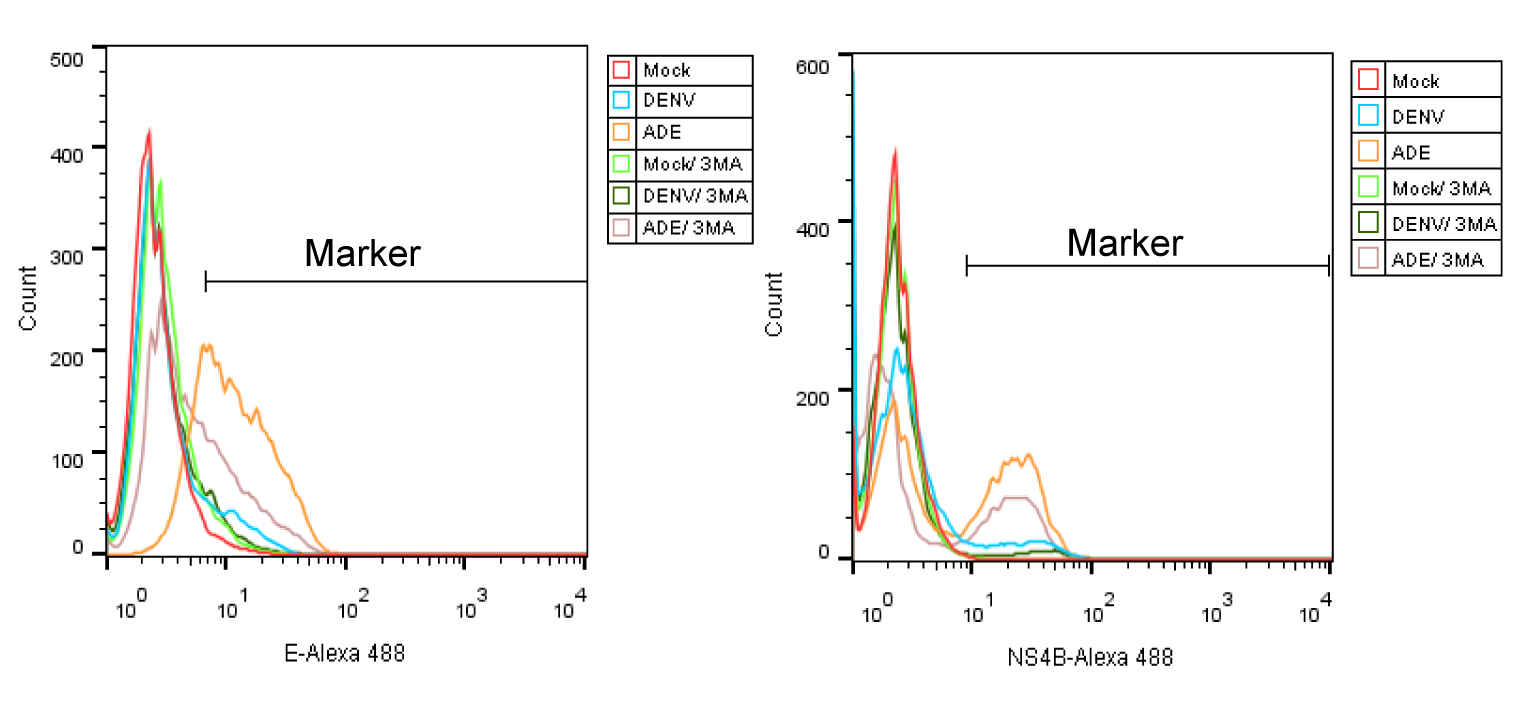

Supplement: Figure S2 — The autophagy inhibitor 3-MA reduces DENV infection. KU812 cells were pre-treated with or without 5 mM 3-MA for 1 h before incubation with medium alone (Mock), DENV alone, or DENV with sub-neutralizing dengue patient sera. 3-MA was maintained in the medium during DENV infection. After 24 h post-infection, the expression of DENV E protein and NS4B protein was detected by flow cytometry. A representative histogram of each group is shown. (TIF) [file pone.0110655.s002.tif]

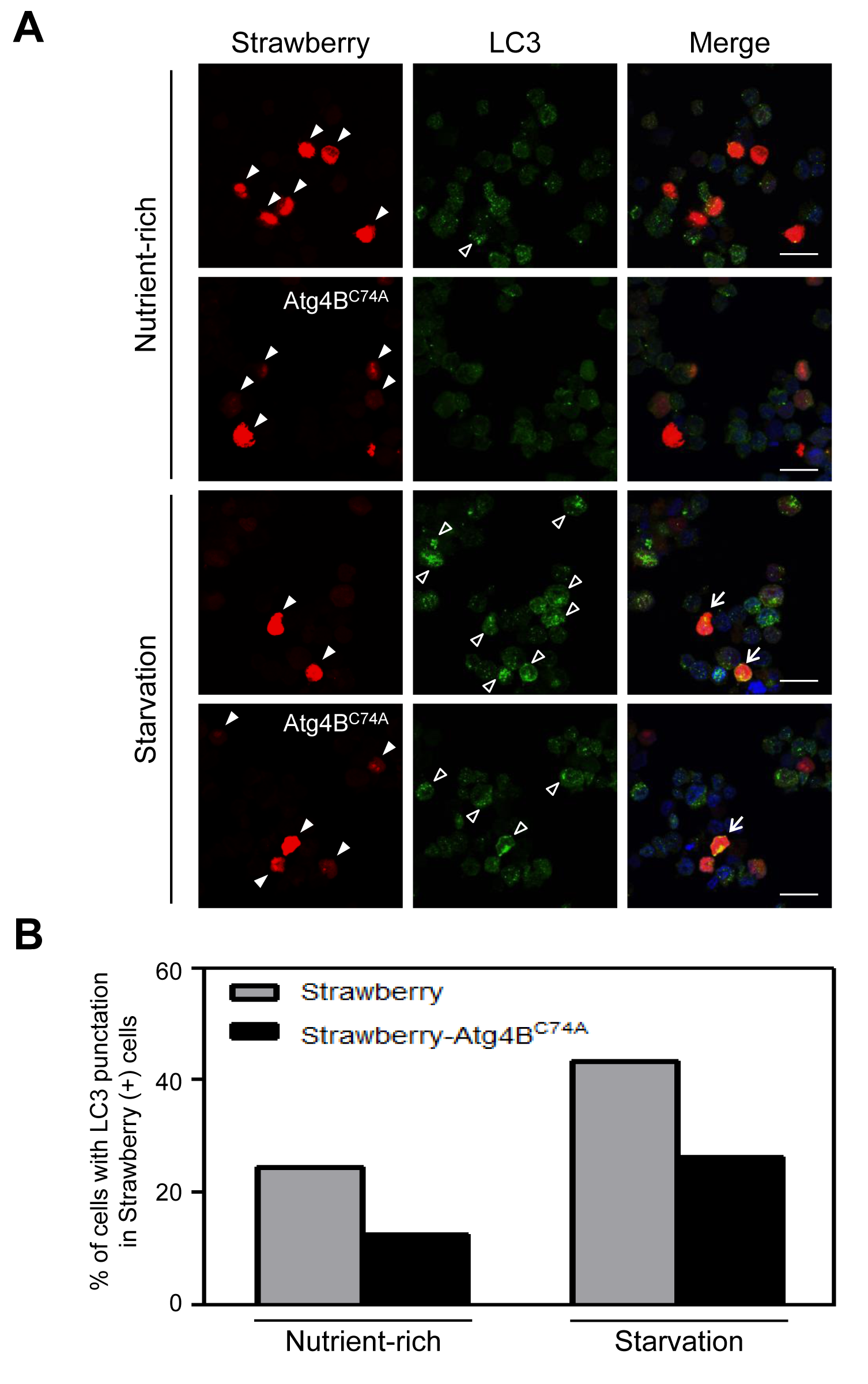

Supplement: Figure S3 — Autophagy is inhibited in the strawberry-Atg4BC74A-expressing KU812 cells. (A) KU812 cells were transfected with strawberry or strawberry-Atg4BC74A plasmids. After transfection and incubation for 48 h, strawberry- and strawberry-Atg4BC74A-expressing KU812 cells were incubated in the nutrient-rich medium or Hank's balanced salt solution (starvation). After 3 h, cells were fixed, permeabilized, stained, and observed by confocal microscopy. The filled arrowheads indicate the strawberry- and strawberry-Atg4BC74A-expressing cells (red). The empty arrowheads indicate LC3 punctation (green). The arrows indicate the cells which possess both green and red fluorescence. The imaging data were repeated two times and one set of representative results is shown. Bar: 20 µm (B) The percentage of LC3 punctation from red cells was quantified from two independent experiments. (TIF) [file pone.0110655.s003.tif]

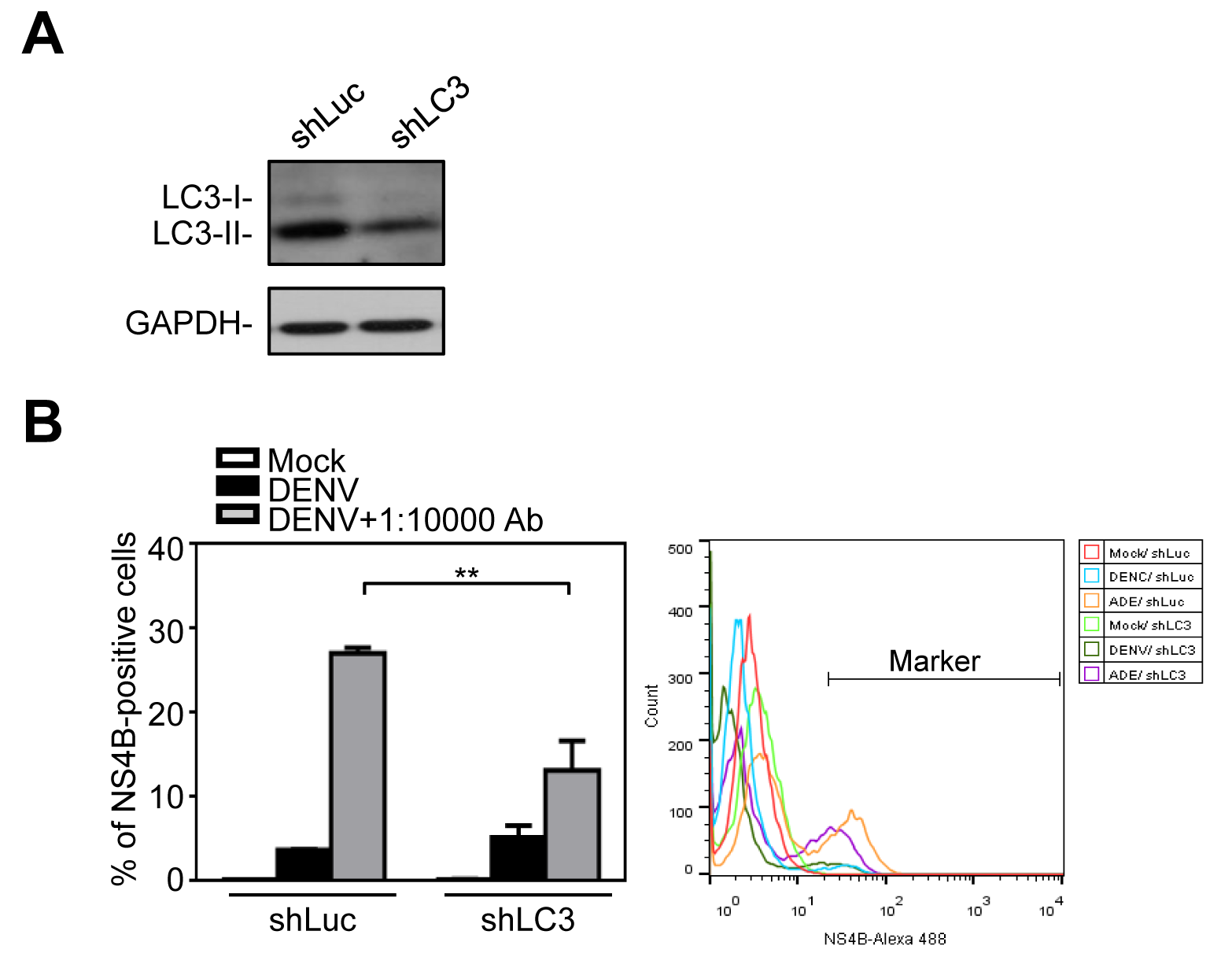

Supplement: Figure S4 — Blockade of LC3 reduces DENV infection. KU812 cells were transfected with shRNA specifically targeting luciferase (shLuc) or LC3 (shLC3). The targeting sequence on luciferase is 5′-GCGCCATTCTATCCGCTGGAA-3′ and the targeting sequence on LC3 is 5′-CGCTTACAGCTCAATGCTAAT-3′. (A) The knockdown efficiency of LC3 in the cells of each group was shown using Western blot analysis. (B) After transfection and incubation for 48 h, cells were infected with DENV alone or DENV with sub-neutralizing dengue patient sera. After 24 h post-infection, the expression of DENV NS4B protein was detected by flow cytometry. A representative histogram of each group is also shown in the right panel. (TIF) [file pone.0110655.s004.tif]
